# Bis({tris[2-(3,5-di-*tert*-butyl-2-oxido­benzylideneamino)ethyl]amine}cerium(III)) diethyl ether solvate

**DOI:** 10.1107/S1600536810039565

**Published:** 2010-10-13

**Authors:** Peter Dröse, Cristian G. Hrib, Frank T. Edelmann

**Affiliations:** a Chemisches Institut der Otto-von-Guericke-Universität, Universitätsplatz 2, D-39116 Magdeburg, Germany

## Abstract

The title compound, 2[Ce(C_51_H_75_N_4_O_3_)]·C_4_H_10_O, was obtained in high yield (92%) by reduction of (TRENDSAL)Ce^IV^Cl [TRENDSAL is *N,N′,N′′*-tris­(3,5-di-*tert*-butyl­salicyl­ide­natoamino)­triethyl­amine] with potassium in THF. The bulky tripodal TRENDSAL ligand effectively encapsulates the central Ce^III^ cation with a Ce—N(imine) distance of 2.860 (2) Å and an average C—N(amine) distance of 2.619 Å within a distorted monocapped octahedral coordination.

## Related literature

For related structures, see: Dröse & Gottfriedsen (2008 ▶); Dröse *et al.* (2010 ▶); Essig *et al.* (2001 ▶); Salehzadeh *et al.* (2005 ▶). In contrast to a previous report (Bernhardt *et al.*, 2001 ▶), reactions of cerium(III) trichloride with either 3,5-di-*tert*-butyl salicylic aldehyde and tris­(2-amino­ethyl­amine) (*in situ* formation of the TRENDSAL ligand) or the free ligand H_3_TRENDSAL afforded only mixtures of Ce(III) and Ce(IV) products. We now found that the trivalent complex can be prepared by reduction of (TRENDSAL)CeCl (Dröse & Gottfriedsen, 2008 ▶) with elemental potassium in THF. 
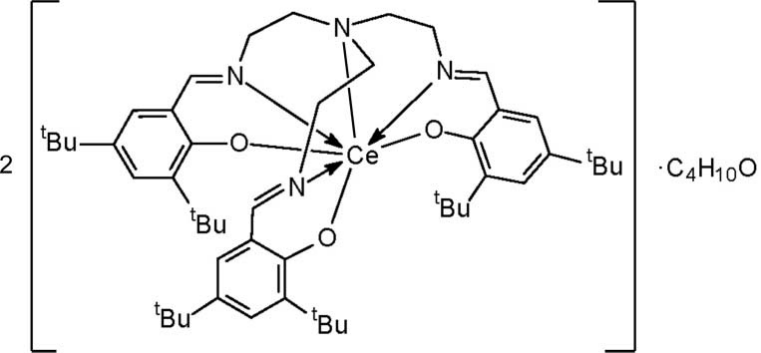


## Experimental

### 

#### Crystal data

2[Ce(C_51_H_75_N_4_O_3_)]·C_4_H_10_O
*M* *_r_* = 1938.66
Monoclinic, 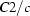

*a* = 27.840 (6) Å
*b* = 16.345 (3) Å
*c* = 24.849 (5) Å
β = 111.39 (3)°
*V* = 10528 (4) Å^3^
*Z* = 4
Mo *K*α radiation
μ = 0.91 mm^−1^
*T* = 180 K
0.45 × 0.34 × 0.33 mm

#### Data collection

STOE IPDS 2T diffractometer
36433 measured reflections
12973 independent reflections
9225 reflections with *I* > 2σ(*I*)
*R*_int_ = 0.050

#### Refinement

*R*[*F* ^2^ > 2σ(*F* ^2^)] = 0.042
*wR*(*F* ^2^) = 0.087
*S* = 1.05
12973 reflections
561 parameters
H-atom parameters constrained
Δρ_max_ = 1.49 e Å^−3^
Δρ_min_ = −0.87 e Å^−3^

### 

Data collection: *X-AREA* (Stoe & Cie, 2002 ▶); cell refinement: *X-AREA*; data reduction: *X-RED32* (Stoe & Cie, 2002 ▶); program(s) used to solve structure: *SHELXS97* (Sheldrick, 2008 ▶); program(s) used to refine structure: *SHELXL97* (Sheldrick, 2008 ▶); molecular graphics: *XP* in *SHELXTL* (Sheldrick, 2008 ▶); software used to prepare material for publication: *SHELXL97*.

## Supplementary Material

Crystal structure: contains datablocks I, global. DOI: 10.1107/S1600536810039565/bt5368sup1.cif

Structure factors: contains datablocks I. DOI: 10.1107/S1600536810039565/bt5368Isup2.hkl

Additional supplementary materials:  crystallographic information; 3D view; checkCIF report

## Figures and Tables

**Fig. 1.**
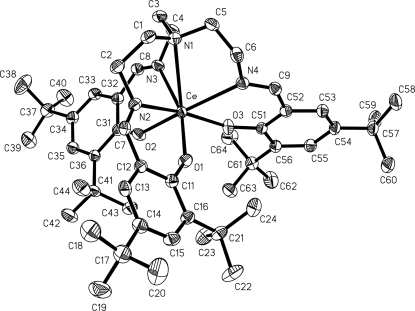
The molecule of the title compound in the crystal. Displacement ellipsoids represent 50% probability levels.

## supplementary crystallographic information

### Comment

The di-*tert*-butyl-substituted heptadentate Schiff-base ligand N[CH_2_CH_2_N=CH-(2-OH-3,5-*^t^*Bu_2_C_6_H_2_)]_3_ (= TRENDSAL) has frequently been employed for rare earth elements such as Ce, Gd, Sm, and Nd, leading in all cases to the formation of mononuclear complexes (Dröse & Gottfriedsen, 2008; Dröse *et al.*, 2010; Essig *et al.*, 2001; Salehzadeh *et al.*, 2005). This very bulky tripodal ligand is generally assumed to encapsulate even the largest lanthanide ions and thereby prevent solvation of the resulting complexes. In contrast to a previous report (Bernhardt *et al.*, 2001), reactions of cerium(III) trichloride with either 3,5-di-*tert*-butyl salicylic aldehyde and tris(2-aminoethylamine) (*in situ* formation of the TRENDSAL ligand) or the free ligand H_3_TRENDSAL afforded only mixtures of Ce(III) and Ce(IV) products. We now found that the trivalent complex can be prepared by reduction of (TRENDSAL)CeCl (Dröse & Gottfriedsen, 2008) with elemental potassium in THF. This new synthetic route afforded pure (TRENDSAL)Ce in excellent yield (92%) in the form of bright orange, air-sensitive crystals. The new compound was fully characterized by elemental analysis and spectroscopic methods. The transition from diamagnetic (TRENDSAL)CeCl to paramagnetic (TRENDSAL)Ce becomes particularly evident in the ^1^H NMR spectra. In the spectrum of (TRENDSAL)Ce the signals are paramagnetically shifted over a range of *ca* 30 p.p.m.. Orange, block-like single crystals were obtained by slow cooling of a saturated solution in diethyl ether to 5 °C.

The coordination geometry around the central cerium(3+) ion can be described as a distorted mono-capped octahedron in which the amine nitrogen (N1) forms the cap. As expected, the overall molecular structure does not differ significantly from those of the previously reported (TRENDSAL)Ln derivatives with Ln = Nd, Sm (Essig *et al.*, 2001) and Ln = Gd (Salehzadeh *et al.*, 2005). Despite the pronounced air-sensitivity of (TRENDSAL)Ce, all attempts to prepare well defined oxidation products, *e.g.* by treatment with Ag[BPh_4_], *p*-benzoquinone, or PhICl_2_, failed.

### Experimental

*Preparation Ce(TRENDSAL)*: A 100 ml Schlenk-flask was charged with 2.93 g (0.58 mol) chloro[*N,N',N''*-tris(3,5-di-*tert*-butylsalicylidenatoamino)triethylamin]cerium(IV) (Dröse & Gottfriedsen, 2008), (= (TRENDSAL)CeCl), and 30 ml of THF and 0.03 g (0.77 mmol, excess) of clean potassium metal pieces were added. Stirring of the reaction mixture for 24 h at r.t. resulted in a color change from purple to orange-yellow. The mixture was evaporated to dryness and the residue was extracted with toluene (20 ml) followed by filtration. The clear filtrate was concentrated *in vacuo* to a total volume of *ca* 5 ml. Cooling to -32 °C for 24 h afforded 0.50 g (92%) of (TRENDSAL)Ce as orange microcrystals. X-ray quality single crystals were grown from a saturated solution in diethyl ether at 5 °C. *M*.p. 143 °C (dec). Anal. calcd for C_51_H_75_CeN_4_O_3_ (932.28 g/mol): C 65.70, H 8.11, N 6.01; found: C 65.47, H 8.04, N 5.63%. IR (KBr pellet): *ν*_max_ 2958 (st, n_s_ CH_3_), 2903 (m, n_as_ CH_2_), 2860 (m, n_as_ CH_3_), 2850 (m, n_s_ CH_2_), 2173 (w), 1622 (*versus*), 1619 (*versus*, C=N), 1615 (*versus*), 1551 (m, C=C Ring), 1535 (st), 1470 (m, d_s_ CH_2_ + d_as_ CH_3_), 1459 (*m*), 1434 (st), 1411 (st), 1391 (st), 1360 (*m*), 1336 (*m*), 1321 (st), 1275 (*m*), 1256 (st, CH ring), 1237 (*m*), 1199 (*m*), 1165 (st), 1138 (w), 1077 (w), 1064 (w), 1037 (w), 1025 (w), 981 (w), 905 (w), 884 (w), 837 (m, CH Ring), 809 (w), 790 (w), 744 (*m*), 735 (w), 698 (w), 641 (w), 614 (w), 588 (w), 555 (w), 541 (w), 523 (w) cm^-1^. ^1^H NMR (400 MHz, THF-d_8_): *d* = 17.99 (s, 3H, –N=C*H*–), 11.61 (s, 3H, Ar–*H*), 9.15 (s, 3H, Ar–*H*), 2.37 (s, 27H,–C(C*H*_3_)_3_), 0.92 (s, 3H, N–CH_2_–C*H*_2_–N=), -1.75 (s, 3H, N–CH_2_–C*H*_2_–N=), -2.16 (s, 27H,–C(C*H*_3_)_3_), -9.71 (s, 3H, N–C*H*_2_–CH_2_–N=), -12.41 (s, 3H, N–C*H*_2_–CH_2_–N=). ^13^C{^1^H} NMR (100.6 MHz, C_6_D_6_, 25 °C): δ = 188.1 (–O–*C*_Ar_), 175.9 (–CH_2_–N=*C*H–Ar), 150.0 (*^t^*Bu–*C*_Ar_), 143.1 (–N=CH–*C*_Ar_), 140.1 (*^t^*Bu–*C*_Ar_), 132.2 (H–*C*_Ar_), 129.3 (H–*C*_Ar_), 40.8 (–CH_2_–*C*H_2_–N=CH–), 35.3 (–*C*H_2_–CH_2_–N=CH–), 33.6 (Ar–*C*Me_3_), 33.5 (–C(*C*H_3_)), 26.7 (–C(*C*H_3_)). EI—MS: *m/z* 931.7 (100) [*M*]^+^, 916.6 (60) [*M* – CH_3_]^+^, 673.3 (45) [*M* – {N(CH_2_)_2_N=CH—Ar}]^+^.

### Refinement

The hydrogen atoms were included using a riding model, with aromatic C—H = 0.95 Å, methyn C—H = 1.00 Å, methylen C—H = 0.99 Å [*U*_iso_(H) = 1.2Ueq(C)] and methyl C—H = 0.98 Å [*U*_iso_(H) = 1.5Ueq(C)].

### Crystal data

**Table:**

2[Ce(C_51_H_75_N_4_O_3_)]·C_4_H_10_O	*F*(000) = 4096
*M**_r_* = 1938.66	*D*_x_ = 1.223 Mg m^−^^3^
Monoclinic, *C*2/*c*	Mo *K*α radiation, λ = 0.71073 Å
*a* = 27.840 (6) Å	Cell parameters from 37307 reflections
*b* = 16.345 (3) Å	θ = 2–28°
*c* = 24.849 (5) Å	µ = 0.91 mm^−^^1^
β = 111.39 (3)°	*T* = 180 K
*V* = 10528 (4) Å^3^	Prism, orange
*Z* = 4	0.45 × 0.34 × 0.33 mm

### Data collection

**Table:**

STOE IPDS 2T diffractometer	9225 reflections with *I* > 2σ(*I*)
Radiation source: fine-focus sealed tube	*R*_int_ = 0.050
graphite	θ_max_ = 28.3°, θ_min_ = 2.1°
Detector resolution: 6.67 pixels mm^-1^	*h* = −37→37
rotation method scans	*k* = −21→21
36433 measured reflections	*l* = −33→33
12973 independent reflections	

### Refinement

**Table:**

Refinement on *F*^2^	Primary atom site location: structure-invariant direct methods
Least-squares matrix: full	Secondary atom site location: difference Fourier map
*R*[*F*^2^ > 2σ(*F*^2^)] = 0.042	Hydrogen site location: inferred from neighbouring sites
*wR*(*F*^2^) = 0.087	H-atom parameters constrained
*S* = 1.05	*w* = 1/[σ^2^(*F*_o_^2^) + (0.0325*P*)^2^ + 19.2734*P*] where *P* = (*F*_o_^2^ + 2*F*_c_^2^)/3
12973 reflections	(Δ/σ)_max_ = 0.001
561 parameters	Δρ_max_ = 1.49 e Å^−^^3^
0 restraints	Δρ_min_ = −0.87 e Å^−^^3^

### Special details

**Table:**

Geometry. All e.s.d.'s (except the e.s.d. in the dihedral angle between two l.s. planes) are estimated using the full covariance matrix. The cell e.s.d.'s are taken into account individually in the estimation of e.s.d.'s in distances, angles and torsion angles; correlations between e.s.d.'s in cell parameters are only used when they are defined by crystal symmetry. An approximate (isotropic) treatment of cell e.s.d.'s is used for estimating e.s.d.'s involving l.s. planes.
Refinement. Refinement of *F*^2^ against ALL reflections. The weighted *R*-factor *wR* and goodness of fit *S* are based on *F*^2^, conventional *R*-factors *R* are based on *F*, with *F* set to zero for negative *F*^2^. The threshold expression of *F*^2^ > σ(*F*^2^) is used only for calculating *R*-factors(gt) *etc*. and is not relevant to the choice of reflections for refinement. *R*-factors based on *F*^2^ are statistically about twice as large as those based on *F*, and *R*- factors based on ALL data will be even larger.

### Fractional atomic coordinates and isotropic or equivalent isotropic displacement parameters (Å^2^)

**Table:**

	*x*	*y*	*z*	*U*_iso_*/*U*_eq_	
Ce	0.329167 (5)	0.498625 (12)	0.474633 (6)	0.01873 (4)	
O1	0.24322 (7)	0.50937 (14)	0.42541 (9)	0.0271 (4)	
O2	0.35362 (8)	0.59761 (14)	0.42595 (10)	0.0267 (5)	
O3	0.34271 (8)	0.38875 (13)	0.42550 (10)	0.0270 (5)	
N1	0.36850 (8)	0.49942 (19)	0.59826 (9)	0.0248 (4)	
N2	0.28670 (9)	0.60565 (15)	0.52270 (10)	0.0241 (5)	
N3	0.42725 (9)	0.53307 (16)	0.52406 (11)	0.0245 (5)	
N4	0.31839 (9)	0.36135 (15)	0.52315 (10)	0.0231 (5)	
C1	0.33944 (12)	0.5602 (2)	0.61895 (13)	0.0275 (6)	
H1A	0.3614	0.5792	0.6581	0.033*	
H1B	0.3086	0.5336	0.6219	0.033*	
C2	0.32275 (12)	0.6335 (2)	0.57898 (14)	0.0283 (7)	
H2A	0.3058	0.6745	0.5955	0.034*	
H2B	0.3532	0.6594	0.5745	0.034*	
C3	0.42411 (11)	0.5221 (2)	0.61973 (13)	0.0288 (7)	
H3A	0.4416	0.4989	0.6588	0.035*	
H3B	0.4274	0.5824	0.6227	0.035*	
C4	0.45041 (11)	0.4907 (2)	0.57957 (12)	0.0288 (6)	
H4A	0.4879	0.5019	0.5964	0.035*	
H4B	0.4453	0.4309	0.5741	0.035*	
C5	0.36233 (12)	0.41688 (19)	0.61908 (13)	0.0275 (6)	
H5A	0.3611	0.4213	0.6583	0.033*	
H5B	0.3926	0.3831	0.6218	0.033*	
C6	0.31350 (13)	0.3746 (2)	0.57939 (14)	0.0270 (7)	
H6A	0.3090	0.3216	0.5963	0.032*	
H6B	0.2830	0.4092	0.5746	0.032*	
C7	0.24086 (11)	0.63523 (17)	0.50472 (13)	0.0233 (6)	
H7	0.2347	0.6776	0.5275	0.028*	
C8	0.45673 (10)	0.57956 (18)	0.50817 (13)	0.0244 (6)	
H8	0.4925	0.5775	0.5311	0.029*	
C9	0.31680 (11)	0.28749 (18)	0.50577 (13)	0.0236 (6)	
H9	0.3095	0.2469	0.5291	0.028*	
C11	0.19961 (10)	0.54758 (17)	0.41628 (12)	0.0220 (5)	
C12	0.19737 (10)	0.61164 (17)	0.45373 (12)	0.0217 (5)	
C13	0.15009 (11)	0.65110 (18)	0.44488 (13)	0.0252 (6)	
H13	0.1490	0.6936	0.4705	0.030*	
C14	0.10574 (11)	0.63007 (19)	0.40052 (13)	0.0259 (6)	
C15	0.10907 (11)	0.5673 (2)	0.36354 (13)	0.0277 (6)	
H15	0.0786	0.5530	0.3321	0.033*	
C16	0.15368 (10)	0.52489 (18)	0.36981 (13)	0.0231 (6)	
C17	0.05388 (12)	0.6731 (2)	0.38862 (16)	0.0344 (7)	
C18	0.05544 (14)	0.7287 (2)	0.43844 (18)	0.0419 (9)	
H18A	0.0649	0.6966	0.4741	0.063*	
H18B	0.0214	0.7534	0.4300	0.063*	
H18C	0.0811	0.7720	0.4433	0.063*	
C19	0.04139 (15)	0.7253 (3)	0.33389 (18)	0.0505 (10)	
H19A	0.0089	0.7545	0.3264	0.076*	
H19B	0.0381	0.6898	0.3010	0.076*	
H19C	0.0692	0.7649	0.3393	0.076*	
C20	0.01111 (15)	0.6101 (3)	0.3802 (2)	0.0587 (13)	
H20A	0.0059	0.5783	0.3451	0.088*	
H20B	−0.0210	0.6383	0.3765	0.088*	
H20C	0.0210	0.5733	0.4136	0.088*	
C21	0.15517 (11)	0.4573 (2)	0.32770 (13)	0.0278 (6)	
C22	0.10220 (13)	0.4425 (3)	0.28058 (16)	0.0455 (9)	
H22A	0.0778	0.4249	0.2984	0.068*	
H22B	0.1051	0.3998	0.2542	0.068*	
H22C	0.0898	0.4932	0.2589	0.068*	
C23	0.19178 (13)	0.4830 (2)	0.29713 (15)	0.0366 (8)	
H23A	0.1923	0.4405	0.2696	0.055*	
H23B	0.2266	0.4904	0.3258	0.055*	
H23C	0.1798	0.5346	0.2765	0.055*	
C24	0.17314 (14)	0.3763 (2)	0.35960 (16)	0.0383 (8)	
H24A	0.1747	0.3344	0.3321	0.057*	
H24B	0.1488	0.3592	0.3776	0.057*	
H24C	0.2075	0.3835	0.3895	0.057*	
C31	0.38962 (10)	0.64423 (17)	0.42079 (12)	0.0209 (5)	
C32	0.44176 (10)	0.63478 (18)	0.45940 (13)	0.0230 (6)	
C33	0.48008 (10)	0.68774 (19)	0.45542 (13)	0.0247 (6)	
H33	0.5147	0.6803	0.4813	0.030*	
C34	0.46936 (11)	0.74999 (19)	0.41545 (13)	0.0255 (6)	
C35	0.41809 (11)	0.75707 (19)	0.37726 (13)	0.0261 (6)	
H35	0.4101	0.7995	0.3492	0.031*	
C36	0.37823 (11)	0.70633 (18)	0.37764 (13)	0.0225 (5)	
C37	0.51156 (11)	0.8114 (2)	0.41629 (15)	0.0315 (7)	
C38	0.52635 (19)	0.8631 (3)	0.4711 (2)	0.0594 (12)	
H38A	0.4956	0.8903	0.4727	0.089*	
H38B	0.5517	0.9044	0.4707	0.089*	
H38C	0.5413	0.8278	0.5050	0.089*	
C39	0.49395 (14)	0.8686 (2)	0.36417 (18)	0.0413 (9)	
H39A	0.5225	0.9047	0.3656	0.062*	
H39B	0.4648	0.9017	0.3649	0.062*	
H39C	0.4832	0.8363	0.3285	0.062*	
C40	0.55927 (13)	0.7654 (3)	0.4160 (2)	0.0466 (10)	
H40A	0.5871	0.8045	0.4201	0.070*	
H40B	0.5508	0.7355	0.3795	0.070*	
H40C	0.5705	0.7267	0.4483	0.070*	
C41	0.32293 (11)	0.71718 (19)	0.33490 (14)	0.0265 (6)	
C42	0.31948 (13)	0.7829 (2)	0.28927 (16)	0.0403 (8)	
H42A	0.3416	0.7673	0.2680	0.060*	
H42B	0.3309	0.8356	0.3085	0.060*	
H42C	0.2837	0.7876	0.2623	0.060*	
C43	0.30158 (14)	0.6380 (2)	0.30144 (16)	0.0395 (8)	
H43A	0.3021	0.5944	0.3287	0.059*	
H43B	0.3230	0.6220	0.2794	0.059*	
H43C	0.2660	0.6470	0.2749	0.059*	
C44	0.28839 (13)	0.7446 (2)	0.36763 (17)	0.0412 (8)	
H44A	0.3021	0.7951	0.3888	0.062*	
H44B	0.2877	0.7017	0.3949	0.062*	
H44C	0.2533	0.7543	0.3400	0.062*	
C51	0.33861 (10)	0.31035 (17)	0.41651 (12)	0.0217 (5)	
C52	0.32485 (10)	0.25835 (18)	0.45449 (13)	0.0232 (6)	
C53	0.32302 (11)	0.17272 (18)	0.44667 (13)	0.0251 (6)	
H53	0.3148	0.1391	0.4733	0.030*	
C54	0.33277 (11)	0.13648 (18)	0.40197 (13)	0.0254 (6)	
C55	0.34397 (11)	0.18913 (18)	0.36348 (13)	0.0262 (6)	
H55	0.3494	0.1652	0.3313	0.031*	
C56	0.34763 (11)	0.27323 (18)	0.36900 (13)	0.0235 (6)	
C57	0.33084 (12)	0.04404 (18)	0.39251 (14)	0.0295 (6)	
C58	0.32820 (14)	−0.0032 (2)	0.44429 (15)	0.0400 (7)	
H58A	0.2967	0.0121	0.4509	0.060*	
H58B	0.3584	0.0101	0.4787	0.060*	
H58C	0.3278	−0.0620	0.4366	0.060*	
C59	0.37920 (14)	0.01503 (19)	0.38204 (18)	0.0412 (9)	
H59A	0.4101	0.0315	0.4147	0.062*	
H59B	0.3799	0.0398	0.3464	0.062*	
H59C	0.3784	−0.0447	0.3783	0.062*	
C60	0.28270 (13)	0.0230 (2)	0.33925 (15)	0.0354 (7)	
H60A	0.2847	0.0509	0.3052	0.053*	
H60B	0.2517	0.0410	0.3458	0.053*	
H60C	0.2811	−0.0362	0.3328	0.053*	
C61	0.36371 (11)	0.32632 (19)	0.32718 (13)	0.0270 (6)	
C62	0.37092 (16)	0.2755 (2)	0.27878 (16)	0.0422 (8)	
H62A	0.3978	0.2343	0.2957	0.063*	
H62B	0.3811	0.3115	0.2533	0.063*	
H62C	0.3384	0.2482	0.2564	0.063*	
C63	0.32315 (13)	0.3919 (2)	0.29867 (15)	0.0349 (7)	
H63A	0.3346	0.4255	0.2729	0.052*	
H63B	0.3186	0.4267	0.3286	0.052*	
H63C	0.2903	0.3656	0.2763	0.052*	
C64	0.41582 (13)	0.3676 (3)	0.36162 (17)	0.0405 (8)	
H64A	0.4411	0.3257	0.3820	0.061*	
H64B	0.4112	0.4058	0.3898	0.061*	
H64C	0.4282	0.3974	0.3350	0.061*	
O4	0.5000	0.5945 (4)	0.2500	0.0883 (17)*	
C93	0.5353 (3)	0.6383 (6)	0.2305 (4)	0.126 (3)*	
H93A	0.5159	0.6757	0.1986	0.151*	
H93B	0.5589	0.6715	0.2625	0.151*	
C94	0.5640 (3)	0.5821 (5)	0.2110 (3)	0.116 (2)*	
H94A	0.5779	0.5392	0.2401	0.174*	
H94B	0.5924	0.6107	0.2048	0.174*	
H94C	0.5415	0.5573	0.1746	0.174*	

### Atomic displacement parameters (Å^2^)

**Table:**

	*U*^11^	*U*^22^	*U*^33^	*U*^12^	*U*^13^	*U*^23^
Ce	0.01725 (6)	0.01654 (6)	0.02120 (7)	0.00030 (7)	0.00557 (4)	0.00044 (8)
O1	0.0187 (8)	0.0272 (12)	0.0316 (10)	0.0048 (9)	0.0047 (7)	−0.0087 (10)
O2	0.0165 (9)	0.0282 (12)	0.0313 (12)	−0.0021 (8)	0.0037 (8)	0.0102 (9)
O3	0.0331 (12)	0.0187 (10)	0.0342 (12)	−0.0025 (9)	0.0183 (10)	−0.0014 (9)
N1	0.0251 (10)	0.0251 (11)	0.0213 (10)	−0.0009 (13)	0.0051 (8)	0.0004 (13)
N2	0.0250 (12)	0.0234 (12)	0.0213 (12)	0.0008 (10)	0.0052 (10)	−0.0030 (10)
N3	0.0193 (11)	0.0238 (11)	0.0255 (12)	0.0017 (9)	0.0023 (9)	0.0057 (10)
N4	0.0247 (12)	0.0231 (12)	0.0218 (12)	0.0006 (10)	0.0090 (9)	0.0003 (10)
C1	0.0275 (14)	0.0312 (16)	0.0216 (14)	−0.0003 (12)	0.0063 (12)	−0.0053 (12)
C2	0.0271 (15)	0.0289 (17)	0.0256 (16)	−0.0010 (13)	0.0056 (12)	−0.0060 (13)
C3	0.0247 (14)	0.0310 (17)	0.0237 (14)	−0.0018 (11)	0.0003 (11)	0.0022 (11)
C4	0.0235 (12)	0.0279 (17)	0.0296 (14)	0.0034 (13)	0.0033 (10)	0.0079 (14)
C5	0.0315 (15)	0.0269 (15)	0.0220 (14)	0.0038 (12)	0.0074 (12)	0.0055 (12)
C6	0.0341 (16)	0.0219 (15)	0.0300 (17)	−0.0003 (12)	0.0176 (14)	0.0023 (13)
C7	0.0269 (14)	0.0183 (13)	0.0247 (14)	0.0016 (11)	0.0093 (11)	−0.0027 (11)
C8	0.0164 (12)	0.0264 (15)	0.0272 (14)	0.0020 (11)	0.0041 (11)	0.0020 (12)
C9	0.0233 (13)	0.0228 (14)	0.0254 (14)	−0.0012 (11)	0.0098 (11)	0.0031 (11)
C11	0.0206 (13)	0.0217 (14)	0.0247 (14)	0.0015 (10)	0.0094 (11)	−0.0002 (11)
C12	0.0205 (12)	0.0212 (14)	0.0221 (13)	0.0017 (10)	0.0061 (10)	−0.0014 (11)
C13	0.0268 (13)	0.0195 (14)	0.0305 (15)	0.0041 (11)	0.0117 (12)	−0.0004 (11)
C14	0.0233 (13)	0.0276 (15)	0.0284 (15)	0.0062 (12)	0.0111 (12)	0.0012 (12)
C15	0.0195 (13)	0.0350 (17)	0.0265 (15)	0.0020 (12)	0.0060 (11)	−0.0024 (13)
C16	0.0188 (12)	0.0254 (14)	0.0245 (14)	−0.0004 (10)	0.0072 (11)	−0.0024 (11)
C17	0.0225 (14)	0.0342 (18)	0.0453 (19)	0.0072 (13)	0.0110 (14)	−0.0027 (15)
C18	0.0328 (17)	0.043 (2)	0.054 (2)	0.0115 (16)	0.0209 (17)	−0.0049 (18)
C19	0.0389 (19)	0.056 (3)	0.046 (2)	0.0221 (18)	0.0029 (17)	0.0016 (19)
C20	0.0282 (19)	0.046 (2)	0.103 (4)	0.0008 (17)	0.024 (2)	−0.018 (2)
C21	0.0197 (13)	0.0354 (18)	0.0274 (15)	−0.0033 (12)	0.0075 (11)	−0.0089 (13)
C22	0.0289 (16)	0.061 (3)	0.040 (2)	−0.0035 (16)	0.0044 (15)	−0.0242 (19)
C23	0.0373 (16)	0.045 (2)	0.0325 (16)	−0.0064 (14)	0.0190 (14)	−0.0111 (14)
C24	0.0422 (18)	0.0296 (17)	0.045 (2)	−0.0042 (15)	0.0174 (16)	−0.0087 (15)
C31	0.0172 (12)	0.0192 (13)	0.0247 (14)	−0.0008 (10)	0.0059 (10)	0.0001 (11)
C32	0.0181 (12)	0.0245 (14)	0.0254 (14)	−0.0009 (11)	0.0068 (11)	−0.0005 (12)
C33	0.0149 (12)	0.0291 (15)	0.0284 (15)	0.0003 (11)	0.0059 (11)	0.0008 (12)
C34	0.0183 (12)	0.0269 (15)	0.0314 (15)	−0.0050 (11)	0.0091 (11)	−0.0016 (12)
C35	0.0241 (13)	0.0251 (15)	0.0281 (15)	−0.0026 (11)	0.0084 (12)	0.0032 (12)
C36	0.0214 (13)	0.0205 (14)	0.0241 (14)	−0.0023 (11)	0.0066 (11)	0.0014 (11)
C37	0.0185 (13)	0.0355 (17)	0.0395 (18)	−0.0082 (12)	0.0093 (12)	0.0014 (14)
C38	0.068 (3)	0.057 (3)	0.057 (3)	−0.038 (2)	0.027 (2)	−0.019 (2)
C39	0.0293 (17)	0.037 (2)	0.058 (2)	−0.0082 (15)	0.0170 (17)	0.0120 (18)
C40	0.0211 (15)	0.050 (2)	0.069 (3)	0.0003 (15)	0.0177 (17)	0.015 (2)
C41	0.0195 (13)	0.0251 (15)	0.0294 (15)	−0.0011 (11)	0.0026 (12)	0.0080 (12)
C42	0.0262 (16)	0.040 (2)	0.046 (2)	−0.0010 (14)	0.0024 (14)	0.0195 (17)
C43	0.0343 (17)	0.0377 (19)	0.0346 (18)	−0.0087 (15)	−0.0017 (14)	0.0015 (15)
C44	0.0260 (15)	0.046 (2)	0.051 (2)	0.0091 (15)	0.0133 (15)	0.0083 (18)
C51	0.0191 (12)	0.0190 (13)	0.0243 (14)	0.0005 (10)	0.0046 (11)	−0.0011 (11)
C52	0.0210 (13)	0.0223 (14)	0.0251 (14)	0.0000 (10)	0.0071 (11)	0.0009 (11)
C53	0.0251 (14)	0.0216 (14)	0.0276 (15)	−0.0023 (11)	0.0083 (12)	0.0010 (12)
C54	0.0251 (14)	0.0192 (14)	0.0309 (15)	−0.0001 (11)	0.0091 (12)	−0.0032 (12)
C55	0.0286 (14)	0.0237 (14)	0.0262 (15)	0.0026 (12)	0.0100 (12)	−0.0034 (12)
C56	0.0213 (13)	0.0223 (14)	0.0268 (15)	0.0001 (11)	0.0085 (12)	−0.0006 (12)
C57	0.0343 (16)	0.0183 (15)	0.0353 (17)	−0.0014 (12)	0.0120 (13)	−0.0032 (12)
C58	0.0568 (19)	0.0215 (14)	0.0407 (17)	0.0005 (19)	0.0165 (15)	0.0009 (19)
C59	0.0398 (18)	0.024 (2)	0.061 (2)	0.0037 (13)	0.0199 (17)	−0.0062 (15)
C60	0.0393 (17)	0.0280 (17)	0.0386 (18)	−0.0020 (13)	0.0138 (15)	−0.0073 (13)
C61	0.0261 (14)	0.0276 (15)	0.0289 (15)	0.0004 (12)	0.0122 (12)	0.0007 (12)
C62	0.059 (2)	0.0370 (19)	0.040 (2)	0.0008 (17)	0.0293 (18)	0.0010 (16)
C63	0.0332 (17)	0.0343 (18)	0.0362 (18)	0.0025 (14)	0.0113 (14)	0.0095 (15)
C64	0.0263 (15)	0.051 (2)	0.046 (2)	−0.0070 (15)	0.0146 (15)	0.0012 (17)

### Geometric parameters (Å, °)

**Table:**

Ce—O1	2.262 (2)	C31—C32	1.427 (4)
Ce—O2	2.268 (2)	C32—C33	1.405 (4)
Ce—O3	2.279 (2)	C33—C34	1.376 (4)
Ce—N3	2.614 (2)	C33—H33	0.9500
Ce—N4	2.616 (2)	C34—C35	1.401 (4)
Ce—N2	2.630 (2)	C34—C37	1.540 (4)
Ce—N1	2.860 (2)	C35—C36	1.388 (4)
O1—C11	1.309 (3)	C35—H35	0.9500
O2—C31	1.302 (3)	C36—C41	1.529 (4)
O3—C51	1.299 (4)	C37—C38	1.525 (5)
N1—C5	1.477 (4)	C37—C39	1.526 (5)
N1—C1	1.487 (4)	C37—C40	1.528 (5)
N1—C3	1.489 (4)	C38—H38A	0.9800
N2—C7	1.283 (4)	C38—H38B	0.9800
N2—C2	1.465 (4)	C38—H38C	0.9800
N3—C8	1.282 (4)	C39—H39A	0.9800
N3—C4	1.466 (4)	C39—H39B	0.9800
N4—C9	1.277 (4)	C39—H39C	0.9800
N4—C6	1.469 (4)	C40—H40A	0.9800
C1—C2	1.516 (5)	C40—H40B	0.9800
C1—H1A	0.9900	C40—H40C	0.9800
C1—H1B	0.9900	C41—C44	1.535 (4)
C2—H2A	0.9900	C41—C43	1.536 (5)
C2—H2B	0.9900	C41—C42	1.539 (4)
C3—C4	1.526 (4)	C42—H42A	0.9800
C3—H3A	0.9900	C42—H42B	0.9800
C3—H3B	0.9900	C42—H42C	0.9800
C4—H4A	0.9900	C43—H43A	0.9800
C4—H4B	0.9900	C43—H43B	0.9800
C5—C6	1.523 (5)	C43—H43C	0.9800
C5—H5A	0.9900	C44—H44A	0.9800
C5—H5B	0.9900	C44—H44B	0.9800
C6—H6A	0.9900	C44—H44C	0.9800
C6—H6B	0.9900	C51—C52	1.423 (4)
C7—C12	1.449 (4)	C51—C56	1.429 (4)
C7—H7	0.9500	C52—C53	1.411 (4)
C8—C32	1.445 (4)	C53—C54	1.371 (4)
C8—H8	0.9500	C53—H53	0.9500
C9—C52	1.452 (4)	C54—C55	1.404 (4)
C9—H9	0.9500	C54—C57	1.527 (4)
C11—C12	1.417 (4)	C55—C56	1.382 (4)
C11—C16	1.424 (4)	C55—H55	0.9500
C12—C13	1.409 (4)	C56—C61	1.540 (4)
C13—C14	1.365 (4)	C57—C58	1.525 (5)
C13—H13	0.9500	C57—C59	1.537 (4)
C14—C15	1.403 (4)	C57—C60	1.540 (5)
C14—C17	1.535 (4)	C58—H58A	0.9800
C15—C16	1.381 (4)	C58—H58B	0.9800
C15—H15	0.9500	C58—H58C	0.9800
C16—C21	1.532 (4)	C59—H59A	0.9800
C17—C18	1.524 (5)	C59—H59B	0.9800
C17—C20	1.530 (5)	C59—H59C	0.9800
C17—C19	1.534 (5)	C60—H60A	0.9800
C18—H18A	0.9800	C60—H60B	0.9800
C18—H18B	0.9800	C60—H60C	0.9800
C18—H18C	0.9800	C61—C63	1.531 (4)
C19—H19A	0.9800	C61—C62	1.534 (4)
C19—H19B	0.9800	C61—C64	1.545 (5)
C19—H19C	0.9800	C62—H62A	0.9800
C20—H20A	0.9800	C62—H62B	0.9800
C20—H20B	0.9800	C62—H62C	0.9800
C20—H20C	0.9800	C63—H63A	0.9800
C21—C24	1.531 (5)	C63—H63B	0.9800
C21—C22	1.531 (4)	C63—H63C	0.9800
C21—C23	1.535 (4)	C64—H64A	0.9800
C22—H22A	0.9800	C64—H64B	0.9800
C22—H22B	0.9800	C64—H64C	0.9800
C22—H22C	0.9800	O4—C93^i^	1.435 (8)
C23—H23A	0.9800	O4—C93	1.435 (8)
C23—H23B	0.9800	C93—C94	1.414 (10)
C23—H23C	0.9800	C93—H93A	0.9900
C24—H24A	0.9800	C93—H93B	0.9900
C24—H24B	0.9800	C94—H94A	0.9800
C24—H24C	0.9800	C94—H94B	0.9800
C31—C36	1.426 (4)	C94—H94C	0.9800
			
O1—Ce—O2	96.91 (8)	H24A—C24—H24C	109.5
O1—Ce—O3	96.93 (8)	H24B—C24—H24C	109.5
O2—Ce—O3	97.81 (8)	O2—C31—C36	121.5 (2)
O1—Ce—N3	162.71 (8)	O2—C31—C32	120.1 (3)
O2—Ce—N3	68.39 (8)	C36—C31—C32	118.4 (2)
O3—Ce—N3	94.17 (8)	C33—C32—C31	119.9 (3)
O1—Ce—N4	92.25 (8)	C33—C32—C8	116.7 (3)
O2—Ce—N4	164.76 (8)	C31—C32—C8	122.9 (2)
O3—Ce—N4	68.91 (8)	C34—C33—C32	122.4 (3)
N3—Ce—N4	104.12 (8)	C34—C33—H33	118.8
O1—Ce—N2	68.39 (8)	C32—C33—H33	118.8
O2—Ce—N2	92.29 (8)	C33—C34—C35	116.7 (3)
O3—Ce—N2	163.18 (8)	C33—C34—C37	120.6 (3)
N3—Ce—N2	102.06 (8)	C35—C34—C37	122.6 (3)
N4—Ce—N2	102.43 (8)	C36—C35—C34	124.4 (3)
O1—Ce—N1	119.69 (7)	C36—C35—H35	117.8
O2—Ce—N1	119.85 (8)	C34—C35—H35	117.8
O3—Ce—N1	120.37 (8)	C35—C36—C31	118.1 (3)
N3—Ce—N1	64.49 (8)	C35—C36—C41	122.1 (3)
N4—Ce—N1	64.68 (8)	C31—C36—C41	119.8 (2)
N2—Ce—N1	64.44 (8)	C38—C37—C39	108.4 (3)
C11—O1—Ce	149.52 (19)	C38—C37—C40	108.9 (3)
C31—O2—Ce	149.49 (19)	C39—C37—C40	108.0 (3)
C51—O3—Ce	148.04 (18)	C38—C37—C34	109.2 (3)
C5—N1—C1	110.0 (2)	C39—C37—C34	112.4 (3)
C5—N1—C3	109.9 (2)	C40—C37—C34	109.8 (3)
C1—N1—C3	109.6 (2)	C37—C38—H38A	109.5
C5—N1—Ce	108.91 (17)	C37—C38—H38B	109.5
C1—N1—Ce	109.33 (17)	H38A—C38—H38B	109.5
C3—N1—Ce	109.07 (16)	C37—C38—H38C	109.5
C7—N2—C2	117.6 (2)	H38A—C38—H38C	109.5
C7—N2—Ce	130.4 (2)	H38B—C38—H38C	109.5
C2—N2—Ce	112.03 (18)	C37—C39—H39A	109.5
C8—N3—C4	117.3 (2)	C37—C39—H39B	109.5
C8—N3—Ce	131.2 (2)	H39A—C39—H39B	109.5
C4—N3—Ce	111.47 (17)	C37—C39—H39C	109.5
C9—N4—C6	117.2 (2)	H39A—C39—H39C	109.5
C9—N4—Ce	130.58 (19)	H39B—C39—H39C	109.5
C6—N4—Ce	112.20 (18)	C37—C40—H40A	109.5
N1—C1—C2	112.1 (2)	C37—C40—H40B	109.5
N1—C1—H1A	109.2	H40A—C40—H40B	109.5
C2—C1—H1A	109.2	C37—C40—H40C	109.5
N1—C1—H1B	109.2	H40A—C40—H40C	109.5
C2—C1—H1B	109.2	H40B—C40—H40C	109.5
H1A—C1—H1B	107.9	C36—C41—C44	109.4 (3)
N2—C2—C1	108.7 (3)	C36—C41—C43	111.8 (3)
N2—C2—H2A	110.0	C44—C41—C43	109.8 (3)
C1—C2—H2A	110.0	C36—C41—C42	111.7 (2)
N2—C2—H2B	110.0	C44—C41—C42	107.7 (3)
C1—C2—H2B	110.0	C43—C41—C42	106.4 (3)
H2A—C2—H2B	108.3	C41—C42—H42A	109.5
N1—C3—C4	111.5 (2)	C41—C42—H42B	109.5
N1—C3—H3A	109.3	H42A—C42—H42B	109.5
C4—C3—H3A	109.3	C41—C42—H42C	109.5
N1—C3—H3B	109.3	H42A—C42—H42C	109.5
C4—C3—H3B	109.3	H42B—C42—H42C	109.5
H3A—C3—H3B	108.0	C41—C43—H43A	109.5
N3—C4—C3	107.8 (2)	C41—C43—H43B	109.5
N3—C4—H4A	110.2	H43A—C43—H43B	109.5
C3—C4—H4A	110.2	C41—C43—H43C	109.5
N3—C4—H4B	110.2	H43A—C43—H43C	109.5
C3—C4—H4B	110.2	H43B—C43—H43C	109.5
H4A—C4—H4B	108.5	C41—C44—H44A	109.5
N1—C5—C6	112.2 (2)	C41—C44—H44B	109.5
N1—C5—H5A	109.2	H44A—C44—H44B	109.5
C6—C5—H5A	109.2	C41—C44—H44C	109.5
N1—C5—H5B	109.2	H44A—C44—H44C	109.5
C6—C5—H5B	109.2	H44B—C44—H44C	109.5
H5A—C5—H5B	107.9	O3—C51—C52	120.2 (3)
N4—C6—C5	108.2 (2)	O3—C51—C56	122.0 (3)
N4—C6—H6A	110.1	C52—C51—C56	117.7 (3)
C5—C6—H6A	110.1	C53—C52—C51	120.4 (3)
N4—C6—H6B	110.1	C53—C52—C9	115.9 (3)
C5—C6—H6B	110.1	C51—C52—C9	123.4 (3)
H6A—C6—H6B	108.4	C54—C53—C52	122.2 (3)
N2—C7—C12	127.7 (3)	C54—C53—H53	118.9
N2—C7—H7	116.1	C52—C53—H53	118.9
C12—C7—H7	116.1	C53—C54—C55	116.5 (3)
N3—C8—C32	127.4 (3)	C53—C54—C57	123.1 (3)
N3—C8—H8	116.3	C55—C54—C57	120.4 (3)
C32—C8—H8	116.3	C56—C55—C54	124.7 (3)
N4—C9—C52	127.5 (3)	C56—C55—H55	117.7
N4—C9—H9	116.2	C54—C55—H55	117.7
C52—C9—H9	116.2	C55—C56—C51	118.4 (3)
O1—C11—C12	119.9 (3)	C55—C56—C61	121.5 (3)
O1—C11—C16	121.2 (2)	C51—C56—C61	120.0 (3)
C12—C11—C16	118.9 (2)	C58—C57—C54	112.4 (3)
C13—C12—C11	119.9 (3)	C58—C57—C59	107.6 (3)
C13—C12—C7	116.7 (3)	C54—C57—C59	110.3 (3)
C11—C12—C7	123.2 (2)	C58—C57—C60	108.5 (3)
C14—C13—C12	121.9 (3)	C54—C57—C60	108.9 (3)
C14—C13—H13	119.0	C59—C57—C60	109.1 (3)
C12—C13—H13	119.0	C57—C58—H58A	109.5
C13—C14—C15	117.2 (3)	C57—C58—H58B	109.5
C13—C14—C17	123.4 (3)	H58A—C58—H58B	109.5
C15—C14—C17	119.4 (3)	C57—C58—H58C	109.5
C16—C15—C14	124.4 (3)	H58A—C58—H58C	109.5
C16—C15—H15	117.8	H58B—C58—H58C	109.5
C14—C15—H15	117.8	C57—C59—H59A	109.5
C15—C16—C11	117.7 (3)	C57—C59—H59B	109.5
C15—C16—C21	122.1 (3)	H59A—C59—H59B	109.5
C11—C16—C21	120.1 (2)	C57—C59—H59C	109.5
C18—C17—C20	107.6 (3)	H59A—C59—H59C	109.5
C18—C17—C19	108.6 (3)	H59B—C59—H59C	109.5
C20—C17—C19	109.9 (4)	C57—C60—H60A	109.5
C18—C17—C14	111.8 (3)	C57—C60—H60B	109.5
C20—C17—C14	110.4 (3)	H60A—C60—H60B	109.5
C19—C17—C14	108.6 (3)	C57—C60—H60C	109.5
C17—C18—H18A	109.5	H60A—C60—H60C	109.5
C17—C18—H18B	109.5	H60B—C60—H60C	109.5
H18A—C18—H18B	109.5	C63—C61—C62	107.6 (3)
C17—C18—H18C	109.5	C63—C61—C56	111.1 (2)
H18A—C18—H18C	109.5	C62—C61—C56	112.2 (3)
H18B—C18—H18C	109.5	C63—C61—C64	109.6 (3)
C17—C19—H19A	109.5	C62—C61—C64	107.8 (3)
C17—C19—H19B	109.5	C56—C61—C64	108.5 (3)
H19A—C19—H19B	109.5	C61—C62—H62A	109.5
C17—C19—H19C	109.5	C61—C62—H62B	109.5
H19A—C19—H19C	109.5	H62A—C62—H62B	109.5
H19B—C19—H19C	109.5	C61—C62—H62C	109.5
C17—C20—H20A	109.5	H62A—C62—H62C	109.5
C17—C20—H20B	109.5	H62B—C62—H62C	109.5
H20A—C20—H20B	109.5	C61—C63—H63A	109.5
C17—C20—H20C	109.5	C61—C63—H63B	109.5
H20A—C20—H20C	109.5	H63A—C63—H63B	109.5
H20B—C20—H20C	109.5	C61—C63—H63C	109.5
C24—C21—C22	107.5 (3)	H63A—C63—H63C	109.5
C24—C21—C16	110.8 (3)	H63B—C63—H63C	109.5
C22—C21—C16	112.2 (3)	C61—C64—H64A	109.5
C24—C21—C23	110.0 (3)	C61—C64—H64B	109.5
C22—C21—C23	107.0 (3)	H64A—C64—H64B	109.5
C16—C21—C23	109.2 (3)	C61—C64—H64C	109.5
C21—C22—H22A	109.5	H64A—C64—H64C	109.5
C21—C22—H22B	109.5	H64B—C64—H64C	109.5
H22A—C22—H22B	109.5	C93^i^—O4—C93	120.1 (9)
C21—C22—H22C	109.5	C94—C93—O4	109.5 (7)
H22A—C22—H22C	109.5	C94—C93—H93A	109.8
H22B—C22—H22C	109.5	O4—C93—H93A	109.8
C21—C23—H23A	109.5	C94—C93—H93B	109.8
C21—C23—H23B	109.5	O4—C93—H93B	109.8
H23A—C23—H23B	109.5	H93A—C93—H93B	108.2
C21—C23—H23C	109.5	C93—C94—H94A	109.5
H23A—C23—H23C	109.5	C93—C94—H94B	109.5
H23B—C23—H23C	109.5	H94A—C94—H94B	109.5
C21—C24—H24A	109.5	C93—C94—H94C	109.5
C21—C24—H24B	109.5	H94A—C94—H94C	109.5
H24A—C24—H24B	109.5	H94B—C94—H94C	109.5
C21—C24—H24C	109.5		
			
O2—Ce—O1—C11	−78.9 (4)	Ce—O1—C11—C16	174.0 (3)
O3—Ce—O1—C11	−177.7 (4)	O1—C11—C12—C13	−178.1 (3)
N3—Ce—O1—C11	−48.1 (5)	C16—C11—C12—C13	0.6 (4)
N4—Ce—O1—C11	113.3 (4)	O1—C11—C12—C7	−3.5 (4)
N2—Ce—O1—C11	10.8 (4)	C16—C11—C12—C7	175.2 (3)
N1—Ce—O1—C11	51.3 (4)	N2—C7—C12—C13	177.0 (3)
O1—Ce—O2—C31	171.1 (4)	N2—C7—C12—C11	2.3 (5)
O3—Ce—O2—C31	−90.9 (4)	C11—C12—C13—C14	−0.6 (4)
N3—Ce—O2—C31	0.5 (4)	C7—C12—C13—C14	−175.6 (3)
N4—Ce—O2—C31	−62.3 (6)	C12—C13—C14—C15	−0.3 (4)
N2—Ce—O2—C31	102.6 (4)	C12—C13—C14—C17	−178.4 (3)
N1—Ce—O2—C31	41.0 (4)	C13—C14—C15—C16	1.3 (5)
O1—Ce—O3—C51	−75.9 (4)	C17—C14—C15—C16	179.5 (3)
O2—Ce—O3—C51	−173.9 (4)	C14—C15—C16—C11	−1.3 (5)
N3—Ce—O3—C51	117.4 (4)	C14—C15—C16—C21	−179.4 (3)
N4—Ce—O3—C51	13.8 (4)	O1—C11—C16—C15	179.0 (3)
N2—Ce—O3—C51	−47.5 (5)	C12—C11—C16—C15	0.4 (4)
N1—Ce—O3—C51	54.6 (4)	O1—C11—C16—C21	−2.9 (4)
O1—Ce—N1—C5	80.51 (19)	C12—C11—C16—C21	178.5 (3)
O2—Ce—N1—C5	−160.35 (16)	C13—C14—C17—C18	−11.6 (5)
O3—Ce—N1—C5	−39.17 (19)	C15—C14—C17—C18	170.3 (3)
N3—Ce—N1—C5	−118.44 (19)	C13—C14—C17—C20	−131.4 (4)
N4—Ce—N1—C5	3.20 (16)	C15—C14—C17—C20	50.5 (4)
N2—Ce—N1—C5	122.56 (18)	C13—C14—C17—C19	108.1 (4)
O1—Ce—N1—C1	−39.7 (2)	C15—C14—C17—C19	−69.9 (4)
O2—Ce—N1—C1	79.43 (19)	C15—C16—C21—C24	−121.6 (3)
O3—Ce—N1—C1	−159.39 (18)	C11—C16—C21—C24	60.4 (4)
N3—Ce—N1—C1	121.3 (2)	C15—C16—C21—C22	−1.4 (4)
N4—Ce—N1—C1	−117.0 (2)	C11—C16—C21—C22	−179.5 (3)
N2—Ce—N1—C1	2.34 (18)	C15—C16—C21—C23	117.1 (3)
O1—Ce—N1—C3	−159.51 (19)	C11—C16—C21—C23	−60.9 (4)
O2—Ce—N1—C3	−40.4 (2)	Ce—O2—C31—C36	−174.5 (3)
O3—Ce—N1—C3	80.8 (2)	Ce—O2—C31—C32	4.4 (6)
N3—Ce—N1—C3	1.54 (19)	O2—C31—C32—C33	−177.0 (3)
N4—Ce—N1—C3	123.2 (2)	C36—C31—C32—C33	2.0 (4)
N2—Ce—N1—C3	−117.5 (2)	O2—C31—C32—C8	−5.4 (4)
O1—Ce—N2—C7	−8.5 (2)	C36—C31—C32—C8	173.6 (3)
O2—Ce—N2—C7	88.0 (3)	N3—C8—C32—C33	171.4 (3)
O3—Ce—N2—C7	−39.0 (4)	N3—C8—C32—C31	−0.5 (5)
N3—Ce—N2—C7	156.4 (3)	C31—C32—C33—C34	0.5 (4)
N4—Ce—N2—C7	−96.0 (3)	C8—C32—C33—C34	−171.6 (3)
N1—Ce—N2—C7	−149.8 (3)	C32—C33—C34—C35	−1.7 (4)
O1—Ce—N2—C2	171.1 (2)	C32—C33—C34—C37	174.3 (3)
O2—Ce—N2—C2	−92.4 (2)	C33—C34—C35—C36	0.5 (5)
O3—Ce—N2—C2	140.6 (3)	C37—C34—C35—C36	−175.5 (3)
N3—Ce—N2—C2	−24.0 (2)	C34—C35—C36—C31	2.0 (5)
N4—Ce—N2—C2	83.6 (2)	C34—C35—C36—C41	179.9 (3)
N1—Ce—N2—C2	29.83 (19)	O2—C31—C36—C35	175.8 (3)
O1—Ce—N3—C8	−39.6 (4)	C32—C31—C36—C35	−3.1 (4)
O2—Ce—N3—C8	−6.4 (3)	O2—C31—C36—C41	−2.1 (4)
O3—Ce—N3—C8	90.3 (3)	C32—C31—C36—C41	179.0 (3)
N4—Ce—N3—C8	159.6 (3)	C33—C34—C37—C38	−66.9 (4)
N2—Ce—N3—C8	−94.1 (3)	C35—C34—C37—C38	108.9 (4)
N1—Ce—N3—C8	−147.9 (3)	C33—C34—C37—C39	172.7 (3)
O1—Ce—N3—C4	139.9 (2)	C35—C34—C37—C39	−11.5 (4)
O2—Ce—N3—C4	173.0 (2)	C33—C34—C37—C40	52.5 (4)
O3—Ce—N3—C4	−90.2 (2)	C35—C34—C37—C40	−131.7 (3)
N4—Ce—N3—C4	−20.9 (2)	C35—C36—C41—C44	−112.8 (3)
N2—Ce—N3—C4	85.4 (2)	C31—C36—C41—C44	65.0 (4)
N1—Ce—N3—C4	31.58 (19)	C35—C36—C41—C43	125.3 (3)
O1—Ce—N4—C9	87.4 (3)	C31—C36—C41—C43	−56.8 (4)
O2—Ce—N4—C9	−39.6 (5)	C35—C36—C41—C42	6.3 (4)
O3—Ce—N4—C9	−9.2 (2)	C31—C36—C41—C42	−175.8 (3)
N3—Ce—N4—C9	−98.2 (3)	Ce—O3—C51—C52	−11.0 (5)
N2—Ce—N4—C9	155.8 (3)	Ce—O3—C51—C56	169.1 (3)
N1—Ce—N4—C9	−150.6 (3)	O3—C51—C52—C53	−176.6 (3)
O1—Ce—N4—C6	−92.6 (2)	C56—C51—C52—C53	3.3 (4)
O2—Ce—N4—C6	140.4 (3)	O3—C51—C52—C9	−2.7 (4)
O3—Ce—N4—C6	170.9 (2)	C56—C51—C52—C9	177.2 (3)
N3—Ce—N4—C6	81.8 (2)	N4—C9—C52—C53	177.4 (3)
N2—Ce—N4—C6	−24.2 (2)	N4—C9—C52—C51	3.2 (5)
N1—Ce—N4—C6	29.44 (18)	C51—C52—C53—C54	−1.9 (4)
C5—N1—C1—C2	−152.9 (2)	C9—C52—C53—C54	−176.3 (3)
C3—N1—C1—C2	86.2 (3)	C52—C53—C54—C55	−1.0 (4)
Ce—N1—C1—C2	−33.3 (3)	C52—C53—C54—C57	−179.8 (3)
C7—N2—C2—C1	118.9 (3)	C53—C54—C55—C56	2.5 (5)
Ce—N2—C2—C1	−60.8 (3)	C57—C54—C55—C56	−178.6 (3)
N1—C1—C2—N2	63.7 (3)	C54—C55—C56—C51	−1.1 (5)
C5—N1—C3—C4	86.2 (3)	C54—C55—C56—C61	175.8 (3)
C1—N1—C3—C4	−152.8 (2)	O3—C51—C56—C55	178.1 (3)
Ce—N1—C3—C4	−33.2 (3)	C52—C51—C56—C55	−1.8 (4)
C8—N3—C4—C3	116.4 (3)	O3—C51—C56—C61	1.1 (4)
Ce—N3—C4—C3	−63.2 (3)	C52—C51—C56—C61	−178.8 (3)
N1—C3—C4—N3	65.0 (3)	C53—C54—C57—C58	−12.0 (4)
C1—N1—C5—C6	85.3 (3)	C55—C54—C57—C58	169.3 (3)
C3—N1—C5—C6	−154.0 (2)	C53—C54—C57—C59	−132.0 (3)
Ce—N1—C5—C6	−34.5 (3)	C55—C54—C57—C59	49.2 (4)
C9—N4—C6—C5	119.6 (3)	C53—C54—C57—C60	108.3 (3)
Ce—N4—C6—C5	−60.4 (3)	C55—C54—C57—C60	−70.5 (4)
N1—C5—C6—N4	64.4 (3)	C55—C56—C61—C63	124.3 (3)
C2—N2—C7—C12	−173.6 (3)	C51—C56—C61—C63	−58.9 (4)
Ce—N2—C7—C12	6.0 (5)	C55—C56—C61—C62	3.8 (4)
C4—N3—C8—C32	−171.8 (3)	C51—C56—C61—C62	−179.3 (3)
Ce—N3—C8—C32	7.6 (5)	C55—C56—C61—C64	−115.2 (3)
C6—N4—C9—C52	−174.6 (3)	C51—C56—C61—C64	61.7 (4)
Ce—N4—C9—C52	5.4 (5)	C93^i^—O4—C93—C94	−177.2 (8)
Ce—O1—C11—C12	−7.3 (5)		

Symmetry codes: (i) −*x*+1, *y*, −*z*+1/2.
